# Excessive Alcohol Consumption, Alcohol Use Disorder and Women

**DOI:** 10.1192/j.eurpsy.2025.121

**Published:** 2025-08-26

**Authors:** R. Agabio, F. Salis

**Affiliations:** Department of Biomedical Sciences, University of Cagliari, Monserrato (CA), Italy

## Abstract

**Abstract:**

Alcohol is a major risk factor for mortality and morbidity worldwide (Agabio et al., 2017). Women are affected by specific alcohol-related consequences, including a dose-dependent increased risk of breast cancer from relatively low levels of alcohol consumption, of which many women remain unaware (Agabio et al., 2022), and risk of foetal alcohol syndrome, in their offspring, if alcohol is consumed during pregnancy (Minozzi et al., 2024). Alcohol use disorder (AUD) is a severe and frequent mental disorder with devasting consequences (Agabio et al., 2017). In US, approximately one out of 4 women suffer for this disorder during their lifetime (MacKillop et al. 2022). Although effective treatments are available (Agabio et al., 2024), AUD is undertreated, with stigma being one of the main reasons for not seeking medical treatment (MacKillop et al. 2022) Women usually experience more severe barriers to AUD treatment than men, with pregnant women experiencing more severe barriers than non-pregnant women (Agabio et al., 2017). Another reason of the scarce use of medical treatment is constituted by the widespread belief that available medications are not effective, or rather, are not effective for all people with AUD. Although sex and gender differences have been described in the response to medications, AUD medications have been studied almost exclusively in men (Agabio et al., 2016). In addition, the number of women with AUD is increasing and services for treatment of AUD should (a) consider women’s specific needs, and (b) realize effective policies to reduce latency prior to accessing medical treatment for both men and women with AUD (Agabio et al., 2021). Nevertheless, recent studies show that only a small number of services have adopted a gender medicine approach in AUD treatment (Vignoli et al., 2024).

**References:**

Agabio et al. Efficacy of Medications Approved for the Treatment of Alcohol Dependence and Alcohol Withdrawal Syndrome in Female Patients: A Descriptive Review. Eur Addict Res. 2016.

Agabio et al. Sex Differences in Alcohol Use Disorder. Curr Med Chem. 2017.

Agabio et al. Gender Differences among Sardinians with Alcohol Use Disorder. J Clin Med. 2021.

Agabio et al. Alcohol Consumption Is a Modifiable Risk Factor for Breast Cancer: Are Women Aware of This Relationship? Alcohol Alcohol. 2022.

Agabio et al. Efficacy of medications for the treatment of alcohol use disorder (AUD): A systematic review and meta-analysis considering baseline AUD severity. Pharmacol Res. 2024.

MacKillop et al. Hazardous drinking and alcohol use disorders. Nat Rev Dis Primers. 2022.

Minozzi et al. Psychosocial and medication interventions to stop or reduce alcohol consumption during pregnancy. Cochrane Database Syst Rev. 2024.

Vignoli et al. Needs of female outpatients with alcohol use disorder: data from an Italian study. Alcohol Alcohol. 2024.

**Disclosure of Interest:**

None Declared

